# SAPHIR: a Shiny application to analyze tissue section images

**DOI:** 10.12688/f1000research.27062.2

**Published:** 2021-04-08

**Authors:** Elodie Germani, Hugues Lelouard, Mathieu Fallet

**Affiliations:** 1 Université de Rennes 1, Rennes, 35000, France; 2 Aix Marseille Univ, CNRS, INSERM, CIML, Marseille, France

**Keywords:** Tissue cellular quantification, spatial cellular profiling, cell-cell interactions, scatterplot, histo-cytometry, image cytometry

## Abstract

Study of cell populations in tissues using immunofluorescence is a powerful method for both basic and medical research. Image acquisitions performed by confocal microscopy notably allow excellent lateral resolution and more than 10 parameter measurements when using spectral or multiplex imaging. Analysis of such complex images can be very challenging and easily lead to bias and misinterpretation. Here, we have developed the Shiny Analytical Plot of Histological Image Results (SAPHIR), an R shiny application for histo-cytometry using scatterplot representation of data extracted by segmentation. It offers many features, such as filtering of spurious data points, selection of cell subsets on scatterplot, visualization of scatterplot selections back into the image, statistics of selected data and data annotation. Our application allows to characterize labeled cells, from their phenotype to their number and location in the tissue, as well as their interaction with other cells. SAPHIR is available from: https://github.com/elodiegermani/SAPHIR

## Introduction

The identification, localization and quantification of cell subsets in tissues is a difficult but essential task for biologists to understand spatial cellular organization in different settings (e.g. homeostasis vs inflammatory diseases or cancer). Advances in optical microscopy allow image acquisitions with more than 10 channel measurements using spectral fluorescence imaging or multiplex imaging combined with z-axis optical slices of tissue sections ranging from 10 µm to more than 200 µm when clearing methods are used ^1– 4^. Analysis of such complex images is very challenging due to the size and complexity of data. It requires image segmentation in 3D that can be further improved using deep learning-based segmentation ^5– 7^. Then, like flow cytometry, complex image analysis can benefit from scatterplot representations that allow to gate cells of interest ^2, 8^. Surprisingly, in existing software, this scatterplot representation is rarely interactive with the image itself, although this would allow to locate selection results back into the image but also to filter or correct results and fine-tune the gates defining cell populations to obtain in return a better visualization of them into the image.

To this end, we developed the Shiny Analytical Plot of Histological Image Results (SAPHIR), an R/Shiny application for the quantitative analysis of tissue section images. Since image segmentation is a complex task in continuous development and highly dependent on image quality and information, the integration of a single type of segmentation method in an image analysis application is not necessarily recommended. Therefore, we decided to separate segmentation from SAPHIR, but we provided in a segmentation menu the link to Fiji and the possibility to run Fiji segmentation macros on user image. Two Fiji macro examples with associated images, which can be used to perform this task before running SAPHIR, are also provided with the application. SAPHIR offers many features such as interactive scatterplots with the image and data filtering and correction as described below.

## Methods

### Data requirement

The segmentation process that is required to use the application SAPHIR was carried out under Fiji ^9^, with homemade macros. These are available on GitHub in the Demonstration Files and can be loaded from the segmentation menu of the application, too. Cell nucleus detection was used for 3D segmentation and the optical section containing the central region of each cell (largest nucleus area) was used to evaluate the fluorescent intensity signal of each channel within a circular ring containing the cell membrane. Finally, the external ring was converted to simple ROI (polygon) and save in .roi for each Cell Of Interest (COI) to be easily readable in R. Each COI was associated with its main optical section (slice). In addition, regions of interest were defined using DBSCAN (optional) ^10^. The segmentation process of the image to be analyzed should create a csv result file containing the intensity values of each channel (ranging from 0 to 255) and, if required, the positioning (x, y, z) linked to each COI. Importantly, many other morphological measurements like area, roundness, or solidity as well as other information (COI belonging to a region of interest, COI interaction with other cells) can be added to the segmentation result file. The result file should contain at least two columns that provide intensities or other parameters necessary to create a scatterplot.

Finally, the minimal requirements to run the application are the image in TIFF with multiple channels and optionally slices, a legend file with all channel information written in column with name headers (Ch1, Ch2,..) and the corresponding labelling (e.g., LT or LB,..), a segmentation result file with intensity values of each COI in csv and the roi.zip file containing contours of each COI. For the TIFF image, since large images may alter the fluidity of the application, we decided that images with a X or Y dimension higher than 1024 would be resized proportionaly so the highest dimension is 1024 (e.g. a 2048 * 1536 image will become a 1024 * 768 image). SAPHIR is able to load standard 2D ROI from Fiji. The segmentation process can be done in either 2D or 3D, but in the latter case only the largest ROI associated with the central slice of each cell is kept. It is possible to convert labelled images (or mask image) in .roi by applying the "analyze particles" function of Fiji or by loading polygon ROI in Fiji and saving them in .roi.

### Operation

SAPHIR was built in the R programming language (version 4.0.2), which has very powerful stastistical packages and excellent interactive dashboard programing package, Shiny and its derivatives like ShinyDashboard or Shinyfiles. Several packages available on CRAN (e.g. ijtiff, magick, ggplot2, plotly) and one package (EBImage) available on Bioconductor were used.

The development of the app was made as follows. First, an interactive Shiny app was created, using the “shiny” library in R, to link a tissue section image and a scatterplot representing for each cell its fluorescence intensity on different channels. To do so, the libraries “ijtiff”, “RImageROI” and “ggplot2” were used. In addition, the statistical part of the app and the “Image to Plot” section were added. Second, the interface of the app was improved with “shinydashboard”, “shinyWidgets” and “shinycssloaders”. Third, a menu “Select your result” was created to load needed files (see Data requirement section). Fourth, the interactivity between the scatter plot and the image was improved using “plotly. A “filtering” option was also added to the scatterplot. Fifth, the visualization options of the scatterplot selections in the image, such as cell ID display, channel overlays and thickness of cells contours were added to the app. This was done using “EBImage” and “magick” packages. Finally, the “Annotation” menu was added to allow result corrections based on scatterplot to image analysis and “Download” buttons were created to allow the download of SAPHIR results.

**Figure 1.**
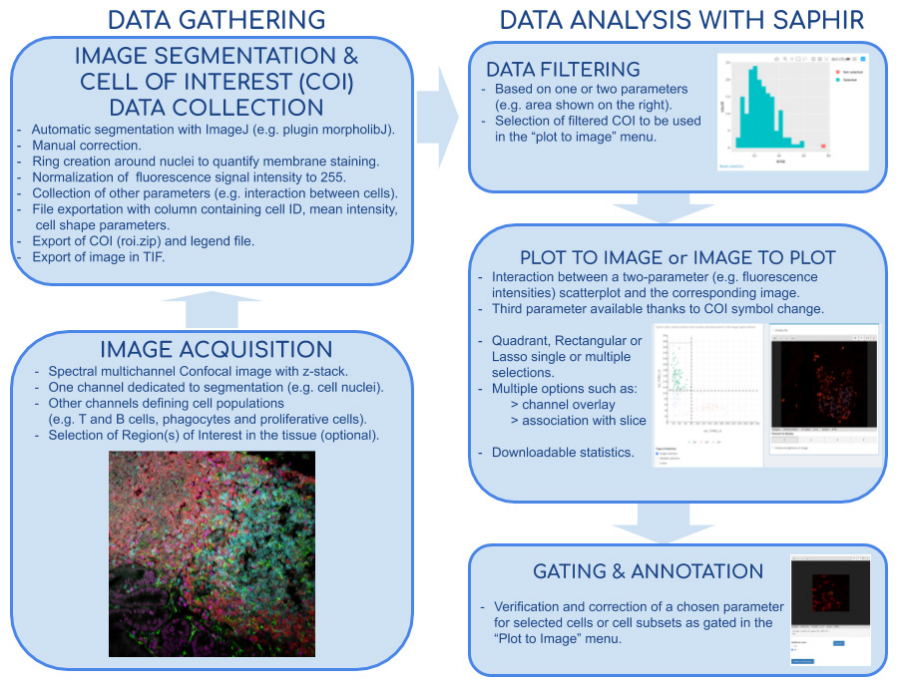
Flow chart of tissue image analysis from image acquisition and segmentation (left side) to extracted data analysis with SAPHIR (right side).

SAPHIR has been tested on macOS and Windows 10. A user flowchart from image acquisition to obtention of quantitative data of COI with SAPHIR is shown in Figure 1.

### Implementation

The first step of SAPHIR is either to run a segmentation program to obtain appropriate files or to load the four required files (image.tif, roi.zip, results.csv, legend.csv) in the “select your results” menu of the application. Here, result, region of interest (ROI) and legend files were obtained with a homemade segmentation macro developed under Fiji as described in the Data requirement section. Fiji and the macro can be launched from the segmentation menu of the application.

**Figure 2.**
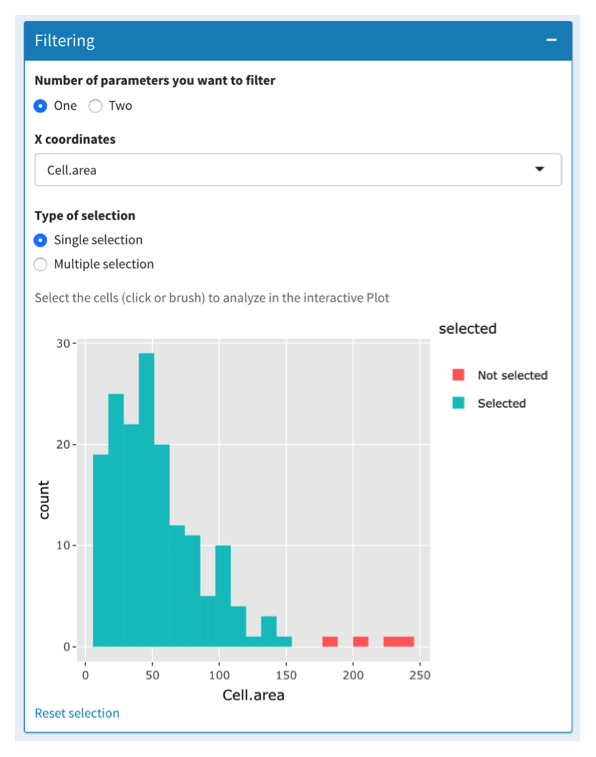
Menu “Plot to Image” of SAPHIR application. The data-filtering tab allows the selection of cells to be analyzed based on one or two parameters. Based on the cell area parameter, only cells that displayed a conventional cell area (in blue) were selected. Badly segmented cells (large area in red) were discarded.

**Figure 3.**
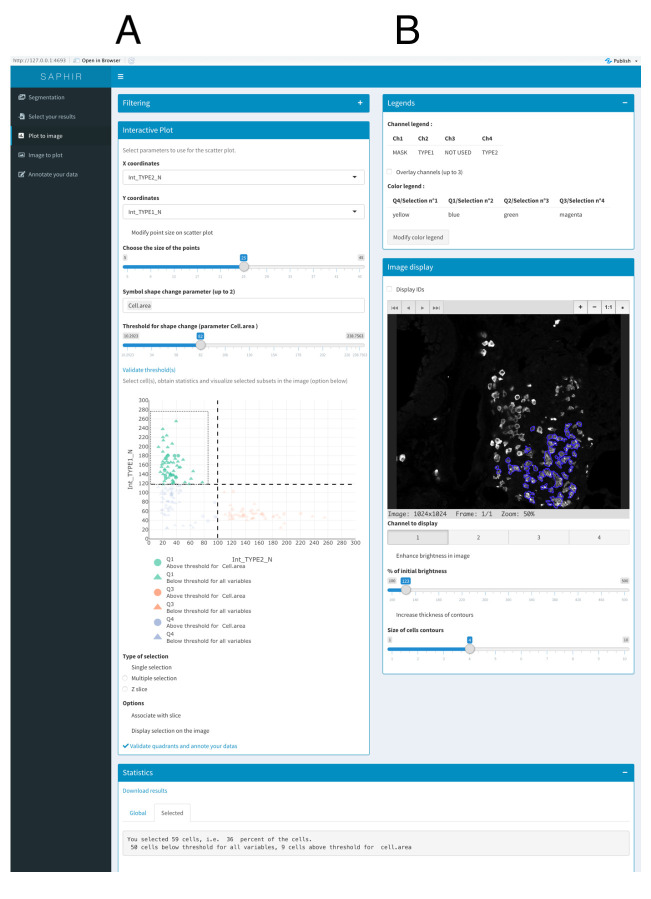
Menu “Plot to Image” of SAPHIR application. ( **A**) Scatterplot of two channel intensity parameters used to gate COI. User can add a third parameter that is displayed on the scatterplot through a change of symbol shape for COI beyond a threshold defined by the user. ( **B**) Visualization of selected cells in the image. Some of the available options are shown (displayed channel(s), channel overlay, cell identity number, brightness).

The first tab of the “Plot to Image” menu is shown in Figure 2 and allows the users to filter their data depending on one or two parameters displayed on histogram or scatterplot, respectively. This can be used to remove cells that have been badly segmented. Indeed, such cells display an aberrant area or volume (doublets or aggregates in red in Figure 2). Here, only cells within the Gaussian curve were retained (in blue in Figure 2). This can also be used to work only on given subpopulations filtered based on their location, interaction with other cells or other criteria (see Use case section below). Then, filtered cells can be separated into subsets thanks to a two-parameter (most often channel intensities) scatterplot ( Figure 3A). In addition, users can add a third parameter that is displayed on the scatterplot through a change of symbol shape for each COI beyond a threshold defined by the users for a given parameter value ( Figure 3A).

Gating can be made easily with quadrants, rectangles and lassos in single or multiple selection mode. Interactivity between scatterplot and image is optional to avoid slow interaction when the size of images exceeds 300 MB, especially using Docker. In the latter case, we recommend users to perform their gating before allowing their selection to be shown in the image by ticking the appropriate box. Users can easily change the slice and the displayed channels with buttons to monitor selected COI spatial distribution and fluorescence intensity in the image ( Figure 3B). Moreover, several options are available such as cell identity display or channel overlay ( Figure 3B). Finally, statistics of the gated COI are downloadable, and gates are saveable for further analyses.

**Figure 4.**
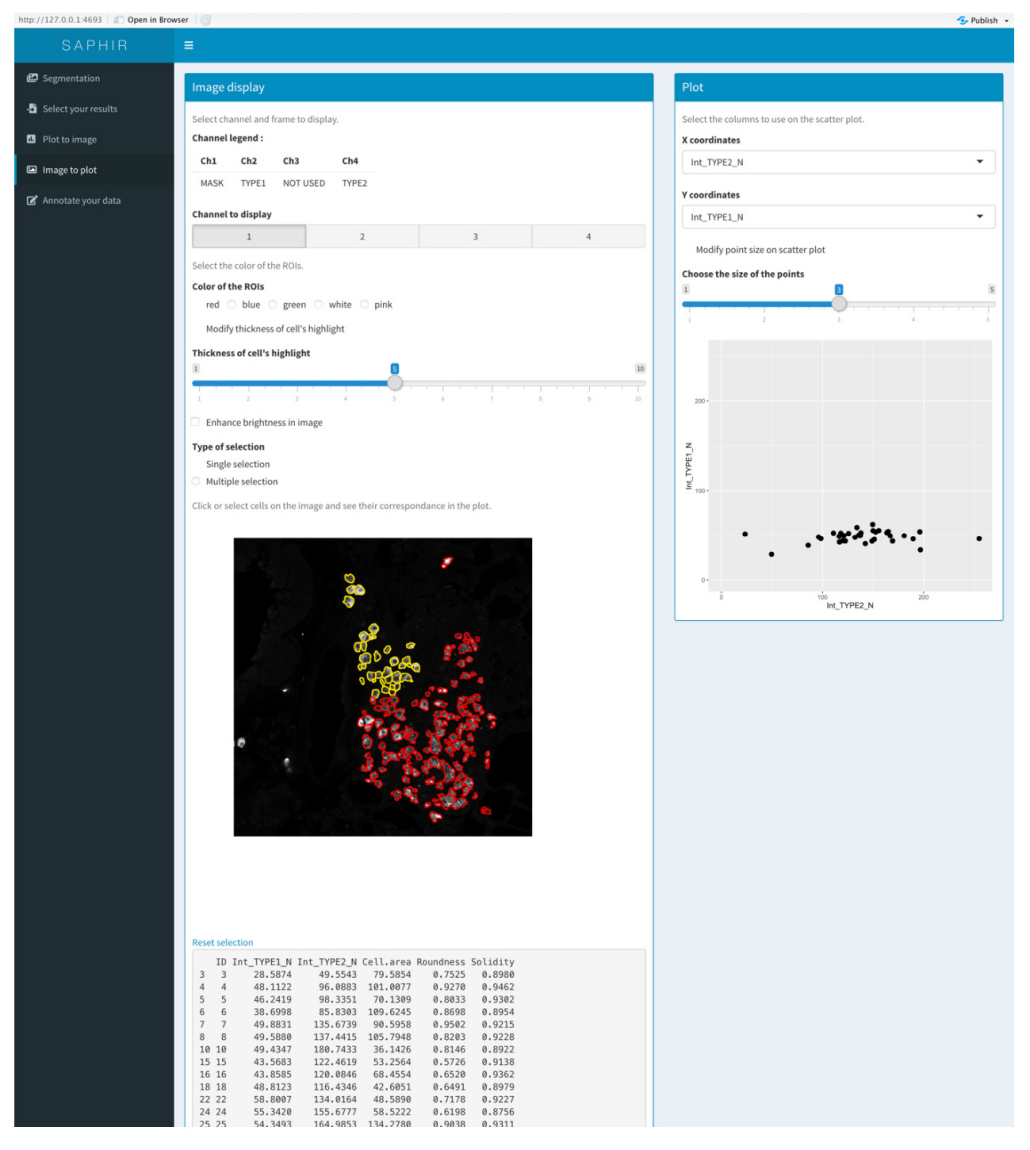
Menu “Image to Plot” of SAPHIR application. Selection of one region in the image (left) and visualization of COI of this region in a corresponding scatterplot where two parameters are displayed (right).

The SAPHIR menu “Image to Plot” allows to select a region in the image and to display cells of this region on a two-parameter scatterplot ( Figure 4).

**Figure 5.**
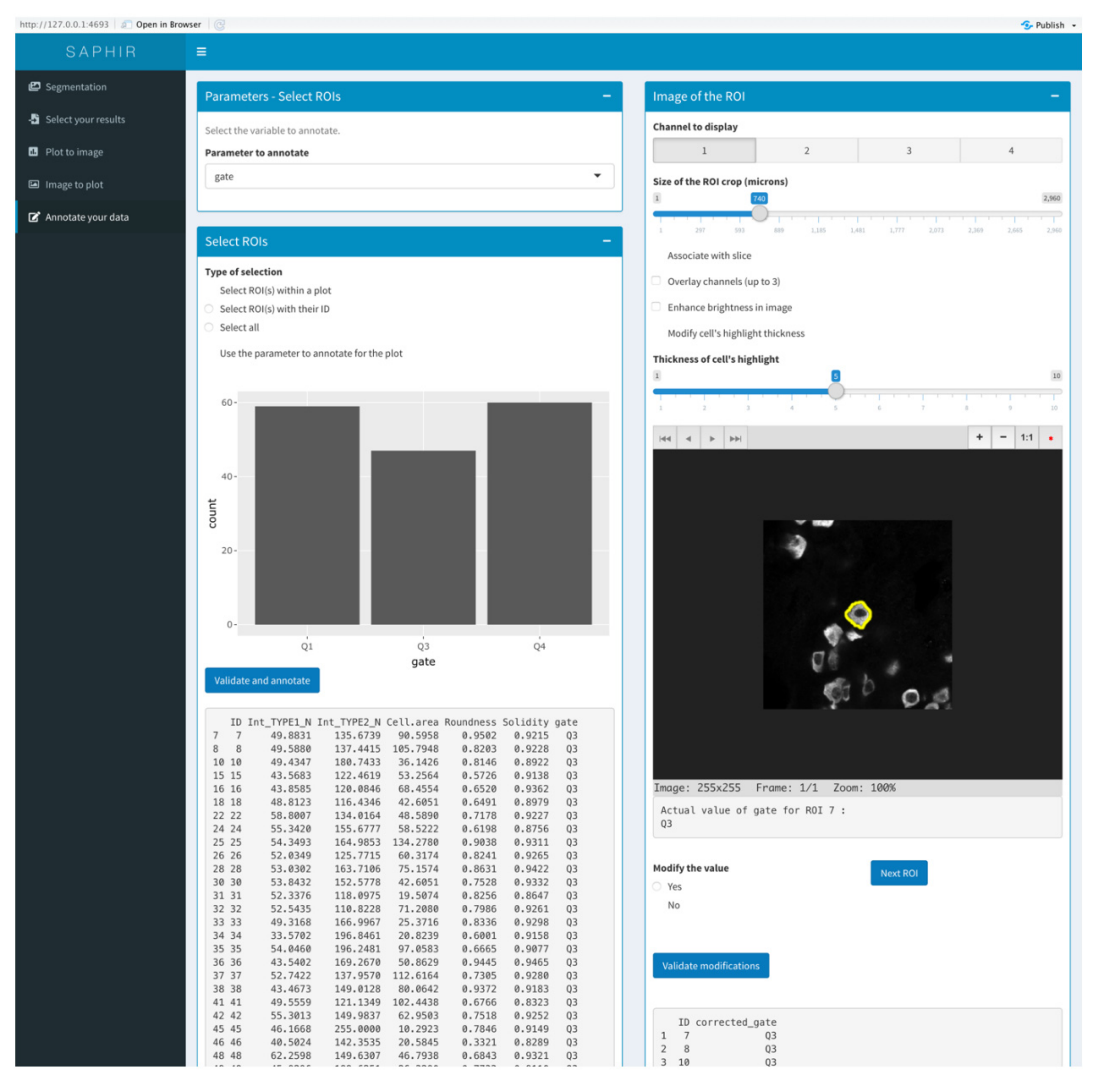
Menu “Annotate your data” of SAPHIR application. The results to annotate can be selected based on the Plot to Image scatterplot gated cells that have been previously saved (left panel). Each cell of the selection can be visualized in the image (right panel) with the possibility to change image size (magnification), the displayed channel(s), the slice (z optical section) and the brightness. Based on the different analysis, results for the selected COI can be modified and final results saved and exported.

Finally, the menu “Annotation” allows correction of the data from the result csv file but also from the scatterplot-gated cells when saved in the “Plot to Image” menu. For each selected cell, a cropped image of this cell is displayed, and based on the previous analyses users can change its parameters (e.g., cell identity) if necessary ( Figure 5).

## Use case

To show the usefulness of our application, we used it on a project that aims to characterize in murine Peyer’s patches (PP) the interaction between phagocytes and proliferative immune effector cells, i.e. B and T cells. PP are immune inductive sites distributed along the small intestine in charge of sampling noxious antigens and mounting an immune response against them. In PP, antigens are taken up by phagocytes, which, upon stimulation, migrate in the T cell zone and its periphery to interact with prime naïve T cells. The periphery of the PP T cell zone is indeed an area of intense proliferation of immune effector cells after stimulation, suggesting that this region is a privileged site for their activation ^11^. We therefore decided to use SAPHIR to examine the evolution of proliferative cell number, to determine their identity (B or T cells) and to analyze their interaction with migratory phagocytes in this region during the course of a stimulation.

We selected a spectral confocal image of a stained stimulated PP. It consisted of 12 optical slices with 6 channels distinguishing proliferative nuclei, B cells, T cells, monocyte-derived phagocytes, early-activated T cells and total nuclei. All optical slices but only the first four channels were necessary for the purpose of this study. First, we used two homemade Fiji macros as described in Data requirement. These macros are available on GitHub in the Demonstration Files and can be launched from the Segmentation menu of the application. The first macro allows the counting of proliferative cells through segmentation of Ki-67 ^+^ nuclei, provides their identity through analysis of T and B cell staining intensity of Ki-67 ^+^ cells, and determines their interaction with phagocytes. The second macro identifies the area of high density in proliferative cells as ROI, thanks to the DBSCAN algorithm ^10^ and determines whether previously analysed cells belong to this ROI. Finally, we obtained a result file that includes 176 COI (proliferative cells of the ROI) analyzed on 10 parameters (the intensity of LT and LB channels; the coordinates x, y, z; 3 physical parameters: area, circularity and solidity; interaction = 1 or not = 0 with a phagocyte; and the belonging = 1 or not = 0 to the region of strong proliferation).

**Figure 6.**
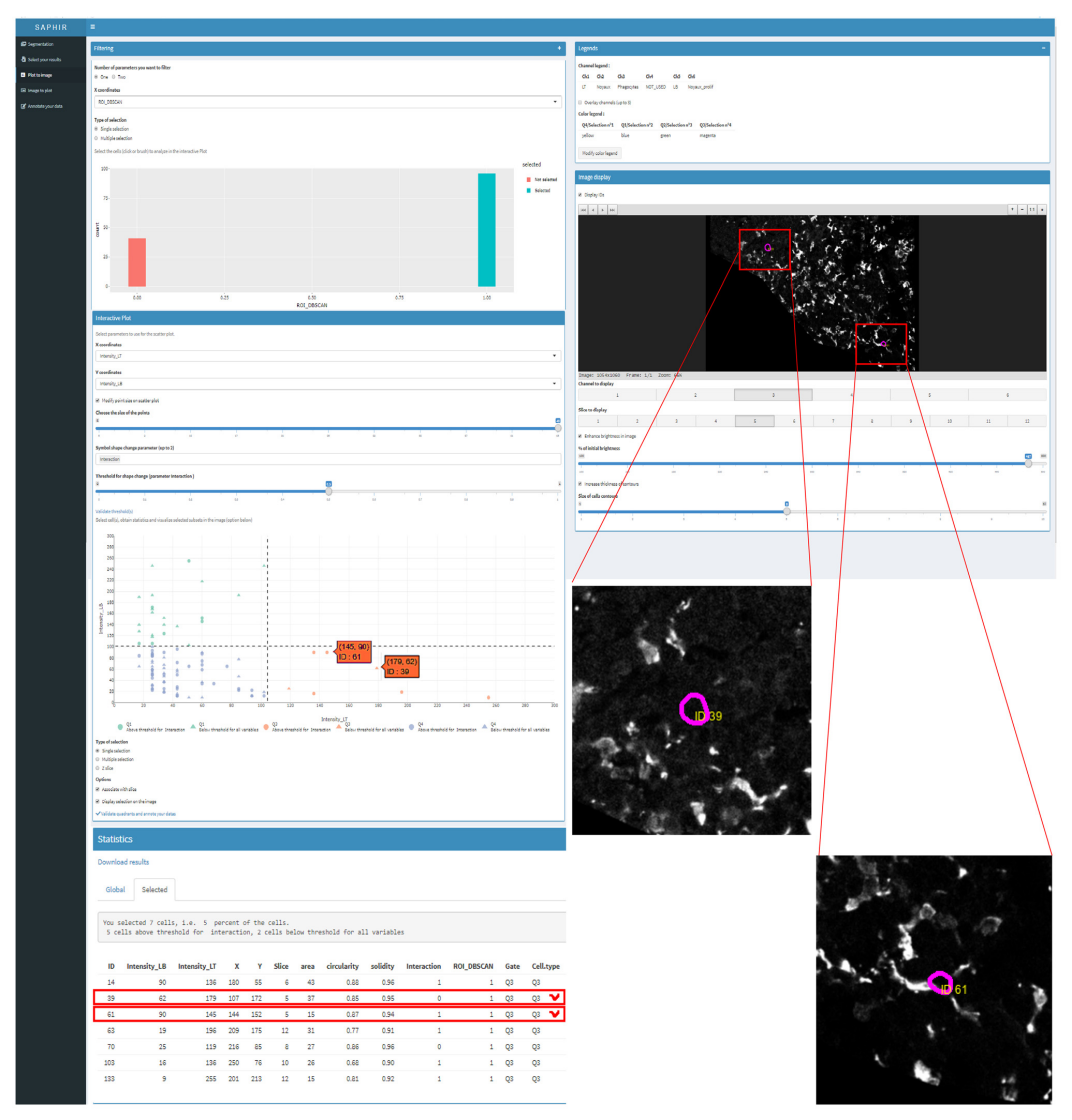
Use case: analysis of proliferative immune effector cells interacting with phagocytes in the T cell zone of murine Peyer’s patches. Upper left: Proliferative cells belonging to the proliferative area of the T cell zone periphery (ROI_DBSCAN) were selected using the Filtering tab (green rectangle). Mid-left: ROI proliferative cells were split into T (orange symbols) and B cells (green symbols) on the scatterplot and phagocyte-interacting cells highlighted (circles) thanks to the symbol shape change parameter. Right: Proliferative T cells selected in the lower left scatter plot were visualized on each optical section (magenta contour). On the shown optical section two proliferative T cells were observed. Higher magnification of the boxed area showed phagocytes (grey cells) interacting with one proliferative T cell (ID 61) but not with the other (ID39). T cell ID numbers allow to locate them back in the scatterplot (pointed out in orange) and in the statistical table (boxed in red) and to check that one but not the other was indeed identified as interacting with phagocytes (circle vs triangle in the scatterplot and 1 vs 0 in the interaction column).

Then, we integrated these data into the SAPHIR application and generated scatterplots and statistical analyses. Thus, with the “Filtering” tab we selected only proliferative cells belonging to the area of intense proliferation at the periphery of the T cell zone from which we wanted to obtain statistical data (green rectangle in the upper left histogram of Figure 6). Then, these ROI-proliferative cells were split into B and T cells using the scatter plot ( Figure 6, left mid-panel). We were thus able to identify proliferative T cells (lower right quadrant, red contour), proliferative B cells (upper left quadrant) and uncharacterized proliferative cells (lower left quadrant). Finally, we used the “symbol shape change parameter” option to highlight ROI-proliferative cells that interacted with phagocytes (circles vs triangles). Selecting T cells, we could localize each of them on the optical slices and check their interaction or not with phagocytes directly into the image ( Figure 6, right panel and higher magnification inserts).

Finally, we obtained exportable statistical data for all gated cells validated or not by direct visualization onto the image ( Figure 6, lower left panel with selected cells boxed in red). In our case, no errors were detected but if they were, the annotation menu would have allowed to correct them. Finally, based on the analysis of this image and several other similar images, we could document the propensity of proliferating T cells to interact with phagocytes at the periphery of the T cell zone upon stimulation (manuscript in preparation).

## Conclusion

The SAPHIR application provides a simple and user-friendly interface to obtain quantitative data from tissue images as well as COI positioning in the tissue. It is based on the interactivity between quantitative data extracted from the image and the image itself. This is especially useful to locate cells that have been selected based on the extracted data. It may thus reveal particular and unsuspected locations for the selected cells within the tissue. It can also be used to check some parameters of the selected cells (e.g., phenotype and interacting partners) on the image to correct any possible errors generated by the automatic analysis (e.g., segmentation and cell identity). Further developments of SAPHIR will include use of pyramidal images to minimize loading and analysis time and will implement R spatial analysis algorithm to analyze cell clusters and cell neighborhood ^12^.

## Data and availability

SAPHIR is provided with segmentation data from two demo pictures ( https://github.com/elodiegermani/SAPHIR/tree/master/Demonstration%20files): one with only two intensity channels and one optical slice to test the application in a very easy and quickly way, and a more complex one with 6 intensity channels, 12 optical slices and two additional parameters (belonging to a ROI and cell-cell interaction) for advanced testing of the application.

These data are parts of several projects conducted in the Center of Immunology of Marseille-Luminy (France). Acquisition of the images was made with spectral confocal microscopy and analyzed with Fiji with the provided macro.

## Software availability

Source code available from: www.github.com/elodiegermani/SAPHIR

Archived source code as at time of publication: https://doi.org/10.5281/zenodo.4656582 ^13^

License: GNU General Public License v3.0
