# Supplementary material for: SAPHIR: a Shiny application to analyze tissue section images
Source: F1000Res. 2021 Apr 8;9:1276. Originally published 2020 Oct 27. [Version 2] doi: 10.12688/f1000research.27062.2 (PMC8078219; doi:10.12688/f1000research.27062.2)
Supplement: Peer review history

# Peer review: Reviewer response for version 2

**DOI:** 10.5256/f1000research.55654.r82892

**Published:** 2021-04-26

**Authors:** Seth Winfree

**Affiliations:** 1 Division of Nephrology, Department of Medicine, Indiana University School of Medicine, Indianapolis, IN, USA

Thank you for taking the time to respond and thoroughly and thoughtfully to my review. I found your responses to my concerns regarding “Rationale for tool development,” “Interpretation of output, results tool performance”, and figure presentation more than adequate. 

 With regard to my minor comments: I believe that addressing bias and inaccuracy is an important and worthwhile pursuit, however, it is unclear how begin able to reclassify cells would specifically address this. In fact, it could be argued that the included functionality could lead to more biased data by allowing for manipulation without clear mechanisms for tracking analysis and changelogs. This appears to be an opportunity for additional contribution in this space (image-based cytometry) and in all multi-step complicated image analysis protocols. How should we qualify segmentation and classification accuracy? However, this is obviously beyond the scope of your work and I would not consider it an impediment for approval. 

 Thank you for taking the time for putting together SAPHIR, I hope to incorporate it into my workflows that, at times, bounce between R and ImageJ/FIJI.

Are the conclusions about the tool and its performance adequately supported by the findings presented in the article?

Partly

Is the rationale for developing the new software tool clearly explained?

Partly

Is the description of the software tool technically sound?

Yes

Are sufficient details of the code, methods and analysis (if applicable) provided to allow replication of the software development and its use by others?

Yes

Is sufficient information provided to allow interpretation of the expected output datasets and any results generated using the tool?

Partly

Reviewer Expertise:

light microscopy, multispectral microscopy, image analysis, tissue cytometry, kidney immunology, neutrophil biology

I confirm that I have read this submission and believe that I have an appropriate level of expertise to confirm that it is of an acceptable scientific standard.

# Peer review: Reviewer response for version 2

**DOI:** 10.5256/f1000research.55654.r82891

**Published:** 2021-04-23

**Authors:** Mahmoud Ahmed

**Affiliations:** 1 Department of Biochemistry and Convergence Medical Sciences, Institute of Health Sciences, Gyeongsang National University School of Medicine, Jinju, South Korea

Based on their response, the modified manuscript, and the updated app, the authors addressed most of the issues raised in the review. These include a description of the app's development process and a detailed interpretation of the results in the use case. A few changes were made in the app itself, including adding a segmentation menu with links to the required macros, a docker file, and a modified script to address the issues with installing and running the app.

 By contrast, the authors made a few arguments and committed to following up with changes to address other issues. Those are

- They explain that the goal of the example use case is to illustrate the functionality rather than provide a real example of the analysis workflow.
- No viable option was provided for users to try/test the app before investing in installing and running it locally. The authors explained that they are working on a strategy to address this issue.
- No substantial improvements to the code were made however the authors committed to cleaning up the code in the future.

Are the conclusions about the tool and its performance adequately supported by the findings presented in the article?

Partly

Is the rationale for developing the new software tool clearly explained?

Yes

Is the description of the software tool technically sound?

Yes

Are sufficient details of the code, methods and analysis (if applicable) provided to allow replication of the software development and its use by others?

Partly

Is sufficient information provided to allow interpretation of the expected output datasets and any results generated using the tool?

Partly

Reviewer Expertise:

image-analysis, genomics, adipocyte-differentiation, autophagy

I confirm that I have read this submission and believe that I have an appropriate level of expertise to confirm that it is of an acceptable scientific standard.

# Peer review: Reviewer response for version 1

**DOI:** 10.5256/f1000research.29894.r73742

**Published:** 2020-11-30

**Authors:** Seth Winfree

**Affiliations:** 1 Division of Nephrology, Department of Medicine, Indiana University School of Medicine, Indianapolis, IN, USA

SAPHIR is an R based tool for image cytometry. SAPHIR provides a novel R based Shinny application for analyzing an existing segmentation, an associated image and measured features of the segmented objects. The tool is intended to meet an unmet need for a flow cytometry-like exploration tool that enables 1) the highlighting of cells-of-interest in the original image volume defined by cells gated on staining intensity or morphological features and 2) the plotting of cells to a scatterplot of staining intensity or morphological features based on an image region-of-interest (ROI). 

**Major criticisms:**

1. Rationale for tool development: A number of tools previously described or referenced in this manuscript include functionality similar to or overlapping with SAPHIR albeit with different implementations. Although the rationale presented by the authors is clearly explained, it is unclear what the rationale is for a new implementation of these functionalities in R (versus CellAnalyst in Python and CodexMAV and Volumetric Tissue Exploration and Analysis in Java). A tool in R could prove very useful especially in light of major R-based projects in transcriptomic analyses and data visualization and the platform's unrivaled strength in statistical analysis, but this is not addressed. Furthermore, R's strengths and weaknesses in imaging (versus other platforms) are not considered or addressed. These points require further justification and discussion. 
2. Rationale for tool development: It is unclear if this tool is designed as a generalizable solution or intended only for ImageJ/FIJI users as it is tied to ImageJ/FIJI functionality to generate a number of input files-especially the segmentation which is based on the ImageJ ROI class. Although there may be a path or utility for using ROIs from Icy, CellProfiler, Ilastik or other common segmentation tools, this is not discussed and needs to be clarified.
3. Interpretation of output results and tool functionality: The authors mention the importance of supporting multiplexed fluorescence images in the introduction, “more than 10 channel measurements”, but in neither the results nor supporting material is it clear to what degree SAPHIR supports these kinds of datasets (e.g. Lelouard *et al.* the manuscript). Furthermore, the support for 3D datasets is unclear, as ImageJ ROIs are limited to 2D and the link referenced in the supporting material for the “Complex Image Analysis”, https://zenodo.org/record/4056001#.X8HVRC2ZM_W, suggests the scripts used to generate the input files only consider a single optical slice. Please clarify these functionalities.
4. Tool performance: The claim of the application’s quickness is hard to assess. Although, SAPHIR appears to function well with the demonstration datasets the authors also qualify their quickness claim: “Interactivity between scatterplot and image is optional to avoid slow interaction when high-resolution images are used. In the latter case, we recommend users to perform their gating before allowing their selection to be shown in the image by ticking the appropriate box.” This apparent contradiction is unqualified, beyond “high-resolution”, the claim of quickness is poorly clarified. Please elaborate, and demonstrate if necessary, this limitation-for instance with larger or more complex datasets.
5. Manuscript: Figures are difficult to interpret due to size and resolution, especially figure 6 in which the “orange” color could not be easily identified nor the “magenta contour”. This is critical to demonstrating the behaviors of SAPHIR. Please adjust to facilitate the interpretation of the results.

**Minor criticisms:**

1. Tool performance: One of the major goals appears to be addressing “bias and misinterpretation” but this is not directly discussed in the results. It is unclear in what way(s) SAPHIR directly addresses bias.
2. Tool performance: The title and introduction suggest that large or mesoscale datasets that can be generated by modern imaging modalities are a unique challenge due to their size and complexity. It is unclear to what degree SAPHIR is able to analyze datasets with a large number of cells. This is an important qualification that is not directly addressed.  

Are the conclusions about the tool and its performance adequately supported by the findings presented in the article?

Partly

Is the rationale for developing the new software tool clearly explained?

Partly

Is the description of the software tool technically sound?

Yes

Are sufficient details of the code, methods and analysis (if applicable) provided to allow replication of the software development and its use by others?

Yes

Is sufficient information provided to allow interpretation of the expected output datasets and any results generated using the tool?

Partly

Reviewer Expertise:

light microscopy, multispectral microscopy, image analysis, tissue cytometry, kidney immunology, neutrophil biology

I confirm that I have read this submission and believe that I have an appropriate level of expertise to confirm that it is of an acceptable scientific standard, however I have significant reservations, as outlined above.

# Response

**Published:** 2021-03-23

**Authors:** Elodie GERMANI

**Affiliations:** University of Rennes 1, France

Dear reviewer, we would like to thank you for all your comments on our app and article that helped us improve them. 

 We studied every remark and wanted to give you an answer : 

**Major criticisms:**

**1. Rationale for tool development: A number of tools previously described or referenced in this manuscript include functionality similar to or overlapping with SAPHIR albeit with different implementations. Although the rationale presented by the authors is clearly explained, it is unclear what the rationale is for a new implementation of these functionalities in R (versus CellAnalyst in Python and CodexMAV and Volumetric Tissue Exploration and Analysis in Java). A tool in R could prove very useful especially in light of major R-based projects in transcriptomic analyses and data visualization and the platform's unrivaled strength in statistical analysis, but this is not addressed. Furthermore, R's strengths and weaknesses in imaging (versus other platforms) are not considered or addressed. These points require further justification and discussion.**

 Due to the short format chosen for our manuscript (allowed initial length of 1000 words), it was not possible to include a discussion on the advantage/disadvantage of each software and its environment. CodexMAV is a commercial software but available for free as an ImageJ plugin. It needs FCS files to run. The software is very powerful to do spatial analysis (t-SNE) of cell populations in tissues, but we didn’t test it in our datasets, yet. CellProfiler (Analyst) is very interesting in term of segmentation and analysis but as far as we know, scatterplots and images are not interconnected. VTEA is also a great and easy application to use, as it is an ImageJ plugin. However, at the time we needed quantitative analysis to be done, segmentation was included in VTEA without all the flexibility that was required for the segmentation of our datasets. We therefore asked the VTEA developer to add the possibility to load ROIs from ImageJ, which is done now. However, the strong interconnection between the image and the scatterplot that we wanted to have to control and correct cells identity was lacking. We have added a sentence in section Operation to explain why we have chosen R for developing SAPHIR application.

**2. Rationale for tool development: It is unclear if this tool is designed as a generalizable solution or intended only for ImageJ/FIJI users as it is tied to ImageJ/FIJI functionality to generate a number of input files-especially the segmentation which is based on the ImageJ ROI class. Although there may be a path or utility for using ROIs from Icy, CellProfiler, Ilastik or other common segmentation tools, this is not discussed and needs to be clarified.**

 Following reviewer recommendation, we have now added a sentence in the data requirement section to help users with the conversion of COI obtained from other software into the Fiji .roi format.

**3. Interpretation of output results and tool functionality: The authors mention the importance of supporting multiplexed fluorescence images in the introduction, “more than 10 channel measurements”, but in neither the results nor supporting material is it clear to what degree SAPHIR supports these kinds of datasets (e.g. Lelouard *et al.* the manuscript). Furthermore, the support for 3D datasets is unclear, as ImageJ ROIs are limited to 2D and the link referenced in the supporting material for the “Complex Image Analysis”, https://zenodo.org/record/4056001#.X8HVRC2ZM_W, suggests the scripts used to generate the input files only consider a single optical slice. Please clarify these functionalities.**

 SAPHIR is supporting multichannel images and Z stack image (ijtiff is used to load the image). We indeed used 3D images but following 3D segmentation only the main COI corresponding to the largest nucleus area was kept for calculating the intensity of other channels. In Fiji, the ROI are linked to specific slices allowing this operation. Thus, we used 2D ROI of 3D images for SAPHIR analysis. This is now explained in the data requirement section.

 Of note, it is now possible to obtain 3D ROI in Fiji using a Plugin from Thomas Boudier ( https://imagejdocu.tudor.lu/plugin/stacks/3d_roi_manager/start#d_roi_manager ).

**4. Tool performance: The claim of the application’s quickness is hard to assess. Although, SAPHIR appears to function well with the demonstration datasets the authors also qualify their quickness claim: “Interactivity between scatterplot and image is optional to avoid slow interaction when high-resolution images are used. In the latter case, we recommend users to perform their gating before allowing their selection to be shown in the image by ticking the appropriate box.” This apparent contradiction is unqualified, beyond “high-resolution”, the claim of quickness is poorly clarified. Please elaborate, and demonstrate if necessary, this limitation-for instance with larger or more complex datasets.**

 As the application could be slowed down with images >300MB, we decided to remove the term "quickly” from the abstract of the manuscript. Moreover, the limitation of 300MB is now specified in the implementation section. Big images (with multiple slices and channels) have to be stored on SSD drive to limit slow interaction. Since our application was mainly used for tissue section < 20 µm, we did not test datasets > 300MB, yet.

**5. Manuscript: Figures are difficult to interpret due to size and resolution, especially figure 6 in which the “orange” color could not be easily identified nor the “magenta contour”. This is critical to demonstrating the behaviors of SAPHIR. Please adjust to facilitate the interpretation of the results.**

 The resolution of the figures has been improved and Figure 6 has been modified to allow better visualization of specific parts of the panels. Moreover, the app tools now include options to adjust the cell contour thickness in the image and the spot size in the scatterplot.

**Minor criticisms:**

**1. Tool performance: One of the major goals appears to be addressing “bias and misinterpretation” but this is not directly discussed in the results. It is unclear in what way(s) SAPHIR directly addresses bias.**

 By allowing to analyze for each cell of the scatterplot its position and its characteristics in the image, SAPHIR allows to check that the parameters obtained automatically with the segmentation and other analysis algorithms fit with the observation of the selected cells in the image. Then, the annotation menu allows to correct any bad analysis (e.g., cell identity). We have now extended the use case section to better describe SAPHIR use.

**2. Tool performance: The title and introduction suggest that large or mesoscale datasets that can be generated by modern imaging modalities are a unique challenge due to their size and complexity. It is unclear to what degree SAPHIR is able to analyze datasets with a large number of cells. This is an important qualification that is not directly addressed.**

 Our aim was clearly not to analyze mesoscale datasets. We apologize if that is the impression given by our title or introduction, although we see no allusion to it in the title and only a small reference in the introduction (i.e., “10 µm to more than 200 µm when clearing methods are used”) but not related to the description of the application. Actually, as mentioned in the introduction our main goal was to obtain an interconnection between the representation of image parameters in scatterplot and the image itself in order to analyze positioning of scatterplot-selected cells in the image and to check and correct any analysis errors by direct observation of the selected cells.

 We hope these responses and the new version of our article will satisfy you.

# Peer review: Reviewer response for version 1

**DOI:** 10.5256/f1000research.29894.r73741

**Published:** 2020-11-11

**Authors:** Mahmoud Ahmed, Trang Huyen Lai

**Affiliations:** 1 Department of Biochemistry and Convergence Medical Sciences, Institute of Health Sciences, Gyeongsang National University School of Medicine, Jinju, South Korea

In this article, the authors describe a shiny app to load and explore the segmentation output of fluorescence images from ImageJ/Fiji. The app provides an interactive interface to filter, select, and annotate the regions of interest as defined by the ImageJ/Fiji macros. Besides, the app allows for performing statistical testing on the selected regions. The manuscript describes the motivation for developing the app, the basic functionality, and a use case. In the use case, the app was used to filter proliferative T and B cells based on cell membranes staining and determine that more T cells interacted with phagocytes in Peyer's patches images.

 The article is written in clear and concise language. The motivation for developing the app and the description of the operation is sound. A few issues remain:

**Major Issues**

1. The implementation section focuses on describing the interface but doesn't explain much about the inner working of the app or the development process. (related, question 3).
2. Four types of files are needed to use the app: Images, Data files, ROIs Files, and Legend files. Each file needs to be processed and prepared to match the formats and requirements of the app. The user has to perform many steps, including using ImageJ and R, to process the files to be able to use SAPHIR. Besides, if the user wants to combine 2 images, he or she has to prepare 4 files for each image. These complicated steps could limit the replication of the analysis by others. It is better if the providers could explain the steps and the tools that they have used to prepare their files. Or if there is an option in SAPHIR that we can use to directly prepare the files.
3. The article explains well using the app to perform different filtering and selection on an example image. However, it doesn't say much about the interpretation of the outputs, or the context in which these decisions could be made. (related, question 4)
4. The use case is very brief and it seems like many of the analysis steps were done outside of the app using custom macros. This makes it hard to evaluate the use case or determining how the app contributed to the final calculations. The authors could provide a more complete description of the use case starting from the raw images and describe how the macros work. It would be helpful to provide links to the data and the tools they used as well.
5. The two examples provided in the article use a single image. To be able to make any claims on the performance (related, question 5), the authors might need to perform testing with multiple images or provide a comparison with existing tools.

**Minor Issues**

1. Several screenshots from the app were included in the manuscript as figures. It might be helpful to change the size/resolution of the images for clarity. Also, highlighting the important parts of the image would make it easier to understand the interface.
2. The app depends on ImageJ so it should be bundled with the app or explain how to obtain and add to the app.
3. Related. Trying to run the app using rstudio server on rocker/verse failed. A dependency libpoppler-cpp-dev was required but not declared.
4. Provide a viable demo, for example using shinyapps.io or rstudio.cloud would make it easier to evaluate the tool and lowers the entry cost for users.
5. The app is written as one long R script. This makes it very difficult to examine, modify, or extend the code. Factoring the code or making it more modular would be helpful.
6. After installing the most recent versions of the required packages, trying to run the app in a fresh rstudio session through an error related to shinyjs::extendShinyjs. I was able to get it to run by adding functions = 'winprint' as an argument. Source

Are the conclusions about the tool and its performance adequately supported by the findings presented in the article?

Partly

Is the rationale for developing the new software tool clearly explained?

Yes

Is the description of the software tool technically sound?

Yes

Are sufficient details of the code, methods and analysis (if applicable) provided to allow replication of the software development and its use by others?

Partly

Is sufficient information provided to allow interpretation of the expected output datasets and any results generated using the tool?

Partly

Reviewer Expertise:

image-analysis, genomics, adipocyte-differentiation, autophagy

We confirm that we have read this submission and believe that we have an appropriate level of expertise to confirm that it is of an acceptable scientific standard, however we have significant reservations, as outlined above.

# Response

**Published:** 2021-03-23

**Authors:** Elodie GERMANI

**Affiliations:** University of Rennes 1, France

Dear reviewer, we would like to thank you for all your comments on our app and article that helped us improve them. 

 We studied every remark and wanted to give you an answer : 

**Major Issues**

**1. The implementation section focuses on describing the interface but doesn't explain much about the inner working of the app or the development process. (related, question 3).**

 Following the recommendation of the reviewer we have now explain the development process of the app in the operation section.

**2. Four types of files are needed to use the app: Images, Data files, ROIs Files, and Legend files. Each file needs to be processed and prepared to match the formats and requirements of the app. The user has to perform many steps, including using ImageJ and R, to process the files to be able to use SAPHIR. Besides, if the user wants to combine 2 images, he or she has to prepare 4 files for each image. These complicated steps could limit the replication of the analysis by others. It is better if the providers could explain the steps and the tools that they have used to prepare their files. Or if there is an option in SAPHIR that we can use to directly prepare the files.**

 We thank the reviewer for this suggestion. We have now added in the data requirement section a description of the segmentation process. A segmentation menu to is available in the application where Fiji and the two macros used in the manuscript can be launched.

**3. The article explains well using the app to perform different filtering and selection on an example image. However, it doesn't say much about the interpretation of the outputs, or the context in which these decisions could be made. (related, question 4)**

 We agree with the reviewer and based on the use case we now describe the results we could obtain and the conclusions we were able to draw using SAPHIR on a specific application, which is the immune response initiation in murine Peyer’s patches. We also show how useful the filtering tool is to focus on a given population of cells (i.e., in our case proliferative cells of the ROI) and how interaction between the image and the scatter plot can be used to focus on a given cell of interest and to control that analysis is correct. 

**4. The use case is very brief and it seems like many of the analysis steps were done outside of the app using custom macros. This makes it hard to evaluate the use case or determining how the app contributed to the final calculations. The authors could provide a more complete description of the use case starting from the raw images and describe how the macros work. It would be helpful to provide links to the data and the tools they used as well.**

 We followed the recommendation of the reviewer and have extended the section dedicated to the use case (see answer to point 3). However, since the segmentation process is now described in detail in the data requirement section, we only briefly described this step to rather focus on the contribution of SAPHIR to quantitative image analysis. As explained in the main text, all data and macros are available on GitHub in the Demonstration Files.

**5. The two examples provided in the article use a single image. To be able to make any claims on the performance (related, question 5), the authors might need to perform testing with multiple images or provide a comparison with existing tools.**

 The main goal of this manuscript was to provide a brief description of the application and its usefulness using two types of image as demos. It is now applied in the lab to study biological processes using dozens of images to validate statistics and its use will be included in several studies, but it was not the aim of this manuscript to already provide this kind of analysis. We indeed tested other tools before developing SAPHIR, such as VTEA and FlowJo, but none was really adapted to our needs as we especially wanted to have a strong interconnection between images and scatterplots.  

**Minor Issues**

**1. Several screenshots from the app were included in the manuscript as figures. It might be helpful to change the size/resolution of the images for clarity. Also, highlighting the important parts of the image would make it easier to understand the interface.**

 The resolution of the figures has been improved and Figure 6 has been modified to allow better visualization of specific parts of the panels. Moreover, the app tools now include options to adjust the cell contour thickness in the image and the size of the dots in the scatterplots.

**2. The app depends on ImageJ so it should be bundled with the app or explain how to obtain and add to the app.**

 Link to Fiji is now provided in the segmentation menu of the app, where Fiji and the two macros used in the manuscript can be launched and applied to any image.

**3. Related. Trying to run the app using rstudio server on rocker/verse failed. A dependency libpoppler-cpp-dev was required but not declared.**

 The dependency to lipoppler-cpp-dev is due to the use of the magick package, which is required to use the application. We also had this problem when trying to deploy our app on shinyapps.io. This problem can be solved using a Unix operating system instead of Windows. We have created a Docker file to use the app without this kind of issues.

**4. Provide a viable demo, for example using shinyapps.io or rstudio.cloud would make it easier to evaluate the tool and lowers the entry cost for users.**

 We are soon going to deploy a demo version on shinyapps.io. However, it will only be operable on our demo images and the user will not be able to change the images to use.

**5. The app is written as one long R script. This makes it very difficult to examine, modify, or extend the code. Factoring the code or making it more modular would be helpful.**

 We agree with the reviewer and we are going to modularize the code in the following months.

**6. After installing the most recent versions of the required packages, trying to run the app in a fresh rstudio session through an error related to shinyjs::extendShinyjs. I was able to get it to run by adding functions = 'winprint' as an argument.** **Source**

 We modified the script in order to correct this. Indeed, the package shinyjs was recently updated and a new argument in the function “extendShinyjs” was made mandatory. We added this modification to the latest version of our script.

 We hope these responses and the new version of our article will satisfy you.
